# Detection of *Alternaria solani* with high accuracy and sensitivity during the latent period of potato early blight

**DOI:** 10.3389/fmicb.2022.1016996

**Published:** 2022-09-23

**Authors:** Zijian Niu, Lijia Zheng, Pan Yang, Jinhui Wang, Mengjun Tian, Yang Pan, Dongmei Zhao, Zhihui Yang, Jiehua Zhu

**Affiliations:** ^1^College of Plant Protection, Hebei Agricultural University, Baoding, China; ^2^Qinhuangdao Plant Protection and Quarantine Station, Qinhuangdao Agricultural and Rural Bureau, Qinhuangdao, China

**Keywords:** detection – plant pathogens, potato, early blight disease, *Alternaria solani*, RNA-based

## Abstract

Early blight (EB) disease, caused mainly by *Alternaria solani*, is an economic threat to potato and tomato production worldwide. Thus, accurate and sensitive detection of the fungal pathogen of this disease in plants at the early infection stage is important for forecasting EB epidemics. In this study, we developed an RNA-based method that enables highly accurate and sensitive *A. solani* detection in a whole potato leaf at a single spore level based on quantitative real-time polymerase chain reaction (qPCR). We discovered jg1677, a highly expressed gene whose full-length coding sequence is very specific for *A. solani*, by analyzing *A. solani* transcripts isolated from enhanced high throughput transcriptome of infected potato leaves by *A. solani* and using the National Center for Biotechnology Information’s basic local alignment search tool. The specificity of the primers derived from jg1677 was determined using 22 isolates of common potato pathogens, including seven *Alternaria* isolates. Detecting jg1677 transcripts with qPCR is 1,295 times more sensitive than detecting genomic DNA. In addition, the expression pattern of jg1677 at different infection stages was determined by qPCR. What is more, jg1677 was expressed relatively stable between 15 and 35°C in infected leaves, and its expression was virtually unaffected in isolated leaves left at room temperature for 24 h. Our work provides a much more sensitive and accurate method compared to conditional DNA-based ones, permitting a very early diagnosis of EB and lowering the risk of EB epidemics.

## Introduction

*Solanum tuberosum* L. (potato) is a widely cultivated food crop in the world, whose production is threatened by various pathogens such as bacteria, viruses, *Oomycetes*, and fungi (Birch et al., 2012; Stokstad, 2019; Campos and Ortiz, 2020). Among these pathogens, *Alternaria solani* is a filamentous fungus that causes the devastating disease, early blight (EB), and leads to significant yield losses in potato crops (Suganthi et al., 2020; Meno et al., 2021, 2022). EB affects plants above ground, with symptoms ranging from small brownish to dark lesions to large ones that always begin on old leaves and grow upward (Sherf and MacNab, 1986; Dhaval et al., 2021). Under ideal conditions, the lesions expand to concentric rings that are regularly surrounded by a yellow halo (Kemmitt, 2002; Bessadat et al., 2017; Dhaval et al., 2021). A shortage of nitrogen supply, increased humidity, appropriate temperature, and rainy weather all increase the possibility of an EB outbreak, of which the occurrence is sudden and on a large scale (Soltanpour and Harrison, 1974; Mamgain et al., 2013; Sahu et al., 2014). Despite the fact that the debris of potato or tomato plants in soil protects *A. solani* from overwintering, this pathogen is aerially transmitted (Rotem, 1994; Chaudhary et al., 2021). Conidia serve as primary inoculum and are spread to the lower leaves by splash, wind, or insects (Rands, 1917; Pandey et al., 2021). Conidia form germ tubes and occasionally invade the host, to which the potato plants are usually resistant when immature but become more susceptible since tuberization (Mamgain et al., 2013; Jindo et al., 2021; Mushrif et al., 2021). Spot-check pathogens on potato leaves will provide timely guidance for EB control after the occurrence of optimal conditions for conidia invasion or post tuber formation (Khan et al., 2018).

Precautionary measures, chemical agent applications, biological control, and the introduction of resistant varieties are options selected for preventing EB in potato crops (Shahbazi et al., 2010; Chowdappa et al., 2013; Devi et al., 2017; Khan et al., 2018). The airborne characteristic of *A. solani* conidium makes it hard to prevent the appearance of this pathogen in potato crops. Crop rotation is time-consuming and cannot meet the needs of modern agriculture (Suganthi et al., 2020; Dhaval et al., 2021; Padol Hrishikesh et al., 2021). Eco-friendly agents, natural extracts, or beneficial bacteria manures have been developed for reducing fungicide usage (Foolad et al., 2008; Mamgain et al., 2013; Kumar et al., 2022; Maurya et al., 2022). In addition, some EB resistance cultivars are introduced (Shahbazi et al., 2010; Odilbekov et al., 2020; Kumari et al., 2021). However, fungicide treatments are still the most effective prophylactic measures, but they are usually rendered ineffective once the disease has begun (Herriott et al., 1986). Thus, developing reliable and sensitive pathogen detection methods is critical for guiding fungicide application in potato crops during the latent period of EB.

*Alternaria solani*, the predominant pathogen of EB disease (on potato and tomato), causes brownish lesions (Adolf et al., 2020; Ivanović et al., 2022). Other *Alternaria* species including *Alternaria grandis*, *Alternaria tenuissima*, and *Alternaria infectoria* are tightly connected with EB, as they can be detected in samples presenting EB symptoms (Rodrigues et al., 2010; Tahery Ardestani et al., 2010; Zheng et al., 2015; Lees et al., 2019). The morphologies of conidia of *Alternaria* species are similar but distinguishable, which makes it possible to identify EB pathogens through macroscopic examination (Rodrigues, 2009; Tymon et al., 2016; Khan et al., 2018). But these plate-based methods are time-consuming and empirical (Khan et al., 2018). So far, new technologies used in EB detection, such as hyperspectral imaging, polymerase chain reactions (PCRs), loop-mediated isothermal amplification (LAMP), real-time quantitative PCR (RT-qPCR), fingerprinting, and sequencing, have emerged, promising more reliable and accurate detection of various plant pathogenic fungi (Bock et al., 2010; Khan et al., 2018; Lees et al., 2019). Wavelength-based hyperspectral images are collected from disease leaves, but this technique cannot be used for disease detection in the early stages of the disease, nor can it distinguish *Alternaria* species in the leaves exhibiting EB symptoms (Leiminger, 2009; Latorse et al., 2010). DNA-based assays have been successfully employed to quantify and distinguish pathogens at subspecies level in soil or host, which has contributed to evaluating the disease severity and guiding disease management (Ling et al., 2010; Duan et al., 2014; Moradi et al., 2014). RT-qPCR is believed the most sensitive method among DNA-based pathogen detection technologies (Ling et al., 2010; Duan et al., 2014; Moradi et al., 2014). Certain transcripts, particularly highly transcribed ones that are repetitively transcribed from the same DNA sequence, are much more abundant than their corresponding DNA under the same conidia condition (Brouwer et al., 2021). In theory, the sensitivities of RNA-based detection methods are far superior to those of DNA-based methods. To ensure PCR specificity and sensitivity, DNA-based primers are typically chosen from the internal transcribed spacers (ITS) regions of nuclear ribosomal DNA (rDNA), making it impossible to detect pathogen RNA transcripts using ITS primers (Chowdappa et al., 2014). To develop *A. solani* RNA-based detection methods, it is necessary to identify *A. solani*-specific genes with high expression levels during infection, which is hampered by a lack of *A. solani* transcription landscape during different infection stages. During the latent period of EB, the number of pathogen cells is much lower than the number of cells in potato leaves, and the transcripts of pathogens and potato leaves are thoroughly blended, resulting in pathogen-derived transcripts being submerged by potato-derived transcripts when RNA sequencing is performed.

When detecting pathogens in a large planted potato fields, we have to detect multiple sampling points mixed to reduce the workload. However, the existing methods cannot guarantee the accuracy as the sample size increases, so there is an urgent need to develop a method that can detect pathogens more sensitively. In our previous study, we increased the depth of RNA sequencing and obtained the transcriptome of spray inoculated potato leaves by *A. solani*, from which the transcripts of *A. solani* were isolated (Wang et al., 2022). In this study, we identified jg1677 in the transcript of *A. solani*, whose full-length sequence is very specific to *A. solani*. The specificity of primers obtained from this transcript was confirmed by using 22 isolates of common potato pathogens based on PCR assay. Moreover, we used quantitative real-time polymerase chain reaction (qPCR) to compare the sensitivity of the RNA-based assay to the DNA-based assay and the stability of jg1677 expression under various conditions. We established a convincing RNA-based method that enables sensitive and accurate pathogen detection during the very early invasion period on potato leaves over a wide range of temperatures.

## Materials and methods

### Source of fungal, oomycetes, and bacterial isolates

The Center of Potato Diseases Research in Hebei Agricultural University provided the 22 strains used in this study, which were isolated and preserved from diseased potatoes collected from various provinces in China. The isolates are *A. solani*, *Alternaria abutilonis*, *Alternaria alternata*, *Fusarium oxysporum*, *Fusarium sambucinum*, *Phytophthora infestans*, *Rhizoctonia solani*, *Streptomyces turgidiscabies*, *Streptomyces stelliscabiei*, *Spongospora subterranea* (Wallr.) Lagerh., *Pectobacterium brasiliense*, *Pectobacterium atrosepticum*, *Pectobacterium versatile*, and *Pectobacterium parvum*.

### DNA extraction

We used the CTAB DNA extraction method in this study. First, the samples were ground to a fine powder and 500 μl CTAB was added, followed by a water bath at 65°C for 10 min. The mixture was then transferred to a new centrifuge tube containing 500 μl of chloroform and inverted several times. Subsequently, spun the centrifuge tube at 12,000 rpm for 10 min, and the upper aqueous phase was transferred into a new microcentrifuge tube, to which 500 μl of chloroform was added. Spun the microcentrifuge tube with the mixture at 12,000 rpm for 10 min, and the upper aqueous phase was transferred into a new microcentrifuge tube again. Added 1 ml of isopropanol and placed at −20°C for 15 min to precipitate DNA. Next, spun the microcentrifuge tube at 12,000 rpm for 10 min at 4°C, resulting in a DNA pellet. The DNA pellet was washed by adding two changes of 75% ethanol to the microcentrifuge tube and then dried at room temperature for 10 min. Finally, dissolved DNA pellet with 30–50 μl ddH_2_O, detected the quality of DNA and stored at 4°C.

### *Alternaria solani* primer design

The *A. solani* transcript sequences were obtained from the National Center for Biotechnology Information’s (NCBI) GenBank database^1^ and one of our previous studies (Wang et al., 2022). The specific primer sets were designed using the Beacon Designer Version 8. All these specific primer sets were synthesized by Sangon Biotech (Shanghai, China) Co., Ltd.

### RNA extraction, DNA digestion, and reverse transcription

The samples we used were *A. solani*-infested potato leaves. The TRIzol RNA extraction method was used in this study and referred to Vidović and Ćuković (2020). We used TransScript One-Step gDNA Removal and cDNA Synthesis Kit/SuperMix by TransGen Biotech for DNA digestion and reverse transcription.

### Sensitivity and specificity detection by quantitative real-time polymerase chain reaction

Quantitative real-time polymerase chain reactions were performed in a CFX96 real-time detection system (Bio-Rad, Hercules, CA, USA) by adding 2 × MagicSYBR Mixture (CoWin Biotech Co., Ltd.) in 96-well, thin-walled, Hard-Shell PCR plate. The reaction mixture comprised 2 × MagicSYBR Mixture (5 μl), 0.5 μl primer (qPCR-F and qPCR-R, 10 mM each), cDNA (4 μl) for a final volume of 10 μl. The amplification program for the reaction was 95°C for 2 min, followed by 40 cycles at 95°C for 5 s, 58°C for 30 s, and 72°C for 20 s, with a fluorescence read at 72°C at the end of each cycle, and a final melting curve at 65–95°C at increments of 0.1°C s^–1^.

## Results

### Determination of the transcript for pathogen detection

**FIGURE 1:**
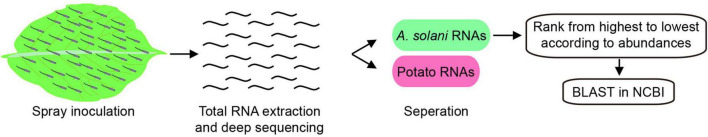
Acquisition workflow of *A. solani*-specific candidate genes. Obtaining total RNA from spray-inoculated potato leaves by *A. solani*, and separating *A. solani* transcripts from total reads. Sorting transcripts from highest to lowest according to their abundances, and BLAST them in NCBI. Finally, verified candidate transcripts.

To create an RNA-based pathogen detection technology, pathogen genes with high expression levels during infection and high specificities for *A. solani* must be identified. The transcripts of *A. solani* that separated from the total reads of infected potato leaves were ranked from highest to lowest according to their abundances. The full-length sequences of the top 10 transcripts (Supplementary Table 1) were first aligned by using the basic local alignment search tool (BLAST) of the NCBI, the flowchart was present in Figure 1. Among the top ten transcripts, jg9669 and gene 02326 were specifically aligned to *A. solani* and jg1677 was aligned to *A. solani* and *Bipolaris maydis*, with alignment identities ranging from 80.77 to 100% (Table 1). This implies that these three transcripts are candidates appropriate for pathogen detection.

**TABLE 1:** Sequences producing significant alignments.

	Description	Scientific name	Max score	Total score	Query cover (%)	*E*-value	Percent identity (%)	Accession length	Accession
jg1677	*Alternaria solani* isolate NL03003 chromosome 5, complete sequence	*Alternaria solani*	185	527	100	2e−42	100	2,771,896	CP022028.1
	*Alternaria solani* isolate NL03003 chromosome 4, complete sequence	*Alternaria solani*	97.1	97.1	32	1e−15	85.71	2,866,555	CP022027.1
	*Bipolaris maydis* ATCC 48331 hypothetical protein partial mRNA	*Bipolaris maydis* ATCC 48331	196	196	90	1e−45	80.77	258	XM014218740.1
	*Bipolaris maydis* clone FNFP45-L01, complete sequence	*Bipolaris maydis*	119	119	28	2e−22	93.67	40,838	AC277244.1
	*Bipolaris maydis* clone FNFP89-G17, complete sequence	*Bipolaris maydis*	119	119	28	2e−22	93.67	33,147	AC277211.1
jg9669	*Alternaria solani* isolate NL03003 chromosome 10, complete sequence	*Alternaria solani*	246	362	96	7e−61	100	1,878,275	CP022033.1
023260	*Alternaria solani* isolate NL03003 chromosome 3, complete sequence	*Alternaria solani*	276	559	100	1e−69	100	3,306,264	CP022026.1

### Specificity of jg1677 derived primers for *Alternaria solani* detection

**FIGURE 2:**
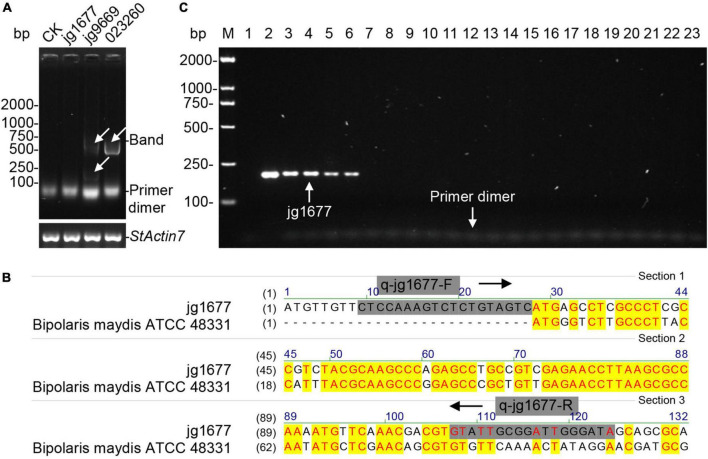
Detection of the specificity of jg1677 primer. **(A)** Agar gel electrophoresis of the PCR products of each primer pair showed no bands for CK and jg1677 and bands for jg9669 and gene 023260. *StActin7* as the loading control. **(B)** The sequence comparison showed that the specific primers q-jg1677-F and q-jg1677-R could not be matched to *Bipolaris maydis* ATCC 48331. **(C)** Detection of jg1677 is *A. solani*-specific. Lane M, DL2000-bp DNA marker; Lane 1, potato cDNA, the negative control; Lane 2–23, the loading order is the same as in Table 2.

The CDS of jg9669, jg1677, and gene 02326 served as candidates for primer design. We chose the recommended pairs of primers automatically designed by Beacon Designer Version 8 software after inputting the CDS sequences of the three genes, respectively (Supplementary Figure 1). Using the DNA of potato leaf as a template, the PCR amplicons of the primers of jg9669 and gene 02326 were detected, indicating that only the primers of jg1677 in the three genes were available for *A. solani* detection (Figure 2A). To avoid interference from *B. maydis*, we used *A. solani*-specific primer pair (Figure 2B). The DNA of five *A. solani* isolates, 2 *Alternaria* subspecies relatives, and 15 other common potato pathogens such as oomycetes, fungi, and bacteria were then used to test the specificity of jg1677 primers (Table 2). The PCR amplicons, 193 bp, of *A. solani* isolates were positively detected, but *A. abutilonis* and *A. alternata* produced no amplicon. As had been expected, *P. infestans*, *F. oxysporum*, *F. sambucinum*, *R. solani*, *S. turgidiscabies*, *S. stelliscabiei*, etc., as well as the negative control neither yield amplification product (Figure 2C). These results illustrated that the jg1677 primer set has a high specificity to *A. solani* and is well suited for pathogen detection on potatoes.

**TABLE 2:** Microbial isolates used in this study.

Species^a^	Host	Code	Source^b^	Primer jg1677^c^
*Alternaria solani*	Potato	HWC-168	HB	+
*Alternaria solani*	Potato	DX12-06	HLJ	+
*Alternaria solani*	Potato	XJ11-17	NX	+
*Alternaria solani*	Potato	WC12-150	HB	+
*Alternaria solani*	Potato	ZB10-46	HB	+
*Alternaria abutilonis*	Abutilon	QY11-19	HB	−
*Alternaria alternata*	Potato	HAUQ-3	HB	−
*Fusarium oxysporum*	Potato	Fo150	IM	−
*Fusarium oxysporum*	Potato	Fo163	HB	−
*Fusarium sambucinum*	Potato	W38-2	IM	−
*Phytophthora infestans*	Potato	JZ13-6	HB	−
*Phytophthora infestans*	Potato	YN1847	YN	−
*Rhizoctonia solani*	Potato	R18	HB	−
*Rhizoctonia solani*	Potato	ZB4	IM	−
*Streptomyces turgidiscabies*	Potato	HY9	SD	−
*Streptomyces stelliscabiei*	Potato	FN1	HB	−
*Spongospora subterranea* (Wallr.) Lagerh.	Potato	FJ-1	HB	−
*Spongospora subterranea* (Wallr.) Lagerh.	Potato	FJ-2	HB	−
*Pectobacterium brasiliense*	Potato	4112	HB	−
*Pectobacterium atrosepticum*	Potato	S1-1	HB	−
*Pectobacterium versatile*	Potato	S111	HB	−
*Pectobacterium parvum*	Potato	S211	HB	−

^a^All isolates of Alternaria spp., fungal and bacterial isolates were collected in Hebei Agricultural University. ^b^The abbreviations are as follows: HB, Hebei Province, China; HLJ, Heilongjiang Province, China; NX, Ningxia Hui Autonomous Region, China; IM, Inner Mongolia Autonomous Region, China; SD, Shandong Province, China. ^c^Note the presence (+) or absence (−) of a PCR amplicon of the expected size.

### Sensitivity of RNA-based pathogen detection method by quantitative real-time polymerase chain reaction

**FIGURE 3:**
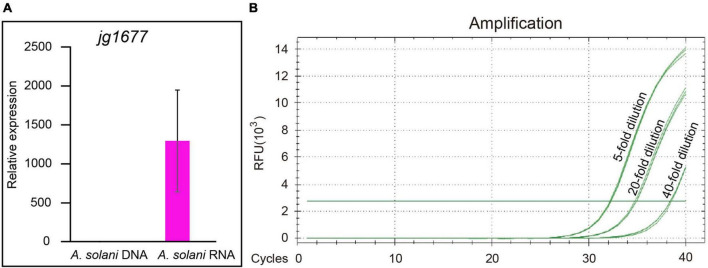
Comparison of the sensitivity of RNA-based and DNA-based detection methods. **(A)** qPCR using DNA and RNA to detect *A. solani* jg1677 expression levels. Data are shown as means ± SD. Error bars represent SD. **(B)** Standard curves for qPCR detection of *A. solani* using single spore cDNA samples diluted fivefold, 20-fold, and 40-fold.

To identify the sensitivity of RNA-based qPCR, a comparison between DNA-based and RNA-based qPCR assays, the most sensitive DNA-based pathogen detection method, was first conducted by using the jg1677 primer pair. The potato leaves infected with *A. solani* were grounded into fine powders with liquid nitrogen and then divided into two equal parts, one for DNA extraction and the other one for RNA extraction. The extracted DNA or RNA was resolved in the same volume of nuclease-free water. In total, 1/10 volume of RNA was reverse transcribed into cDNA by jg1677 specific primer. A half of cDNA and 1/20 volume of DNA served as templates for qPCR using the jg1677 primer pair. The products length of the two were similar, 116 and 193 bp, respectively, both of which were suitable for qPCR and transcript of potato *Actin 7* serves as the loading control. The RNA-based pathogen detection method outperforms the DNA-based method by 1,295-fold by using samples cultured at 25°C (Figure 3A). Next, we inoculated the potato leaves with one spore, used the microscope to determine its germination, and conducted the qPCR assay after 24 h. The total RNA of infected leaves was extracted, 1/5 of the total RNA was reverse transcribed into cDNA by gene-specific primer, and the cDNA was diluted 5-, 20-, and 40-fold, respectively, and then the qPCR assay was performed. The Ct values of 5- and 20-times diluted samples were 32.3 and 34.8, both of which were credible and indicated the RNA-based method promised EB detection at a single spore level (Figure 3B). Therefore, we greatly improved the sensitivity of RNA based detection method compared to DNA based method. So, RNA-based detection with the jg1677 primer can significantly improve pathogen detection sensitivity.

### Profiling of the transcription pattern of jg1677

**FIGURE 4:**
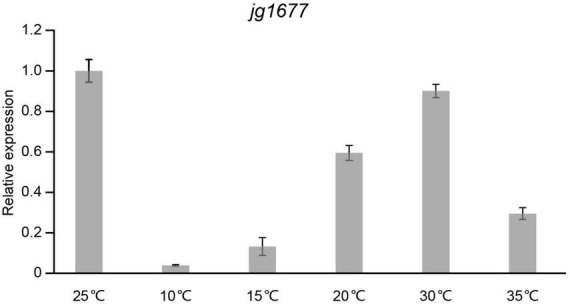
Expression of jg1677 at different temperatures. qPCR detection of jg1677 expression at different temperatures. Data are shown as the expression of jg1677 compared to that at 25°C. Error bars represent SD.

Temperature is one of the main factors that influence the expression level of pathogen genes on potato leaves in the field. To assess the expression levels of jg1677 on potato leaves infected by *A. solani* at different temperatures, we performed qPCR assays with leaves treated at temperatures ranging from 10 to 35°C. The expression of jg1677 increased from 10 to 25°C, with the highest expression at 25°C, and then decreased from 25 to 35°C. The expression at 10°C is 3.92% of that at 25°C, 15°C is 13.25% of that at 25°C, 20°C is 59.48% of that at 25°C, 30°C is 90.07% of that at 25°C, and 35°C is 29.52% of that at 25°C (Figure 4). Given that the sensitivity of the RNA-based assay is more than a thousand times greater than that of the DNA-based assay, and despite the fact that jg1677 expression at 15°C is 13.25% of that at 25°C, we believe that jg1677 expression at 15°C is sufficient to be detected. These findings showed that RNA-based pathogen detection is applicable across a wide temperature range.

**FIGURE 5:**
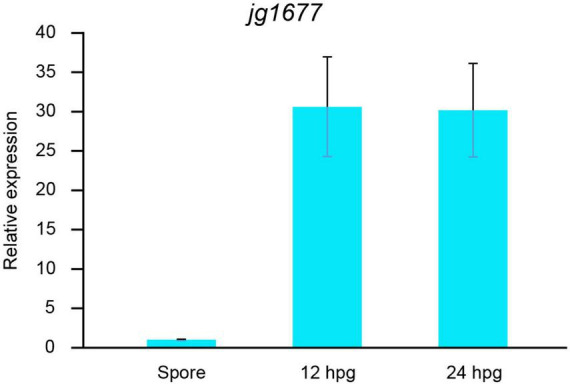
Expression levels of jg1677 before and post spore germination. qPCR detection of jg1677 expression at different germination periods. Data are shown as the expression of jg1677 compared to conidia. Error bars represent SD.

We also profiled the expressions of jg1677 at different stages of the life cycle of *A. solani*. The results showed that, while the jg1677 expression level was low in the conidia, it was significantly higher at 12 h post spore germination, 30-fold higher than in the spores, and stable high expression at the following 12 h (Figure 5). These findings indicated that jg1677 is available to detect pathogens at different infection conditions on potato leaves. During the developmental cycle of *A. solani*, jg1677 showed high expression at all stages except for the low expression of jg1677 in spores, indicating its suitability for pathogen detection.

### Stability of jg1677 transcript from detached potato leaves infected by *Alternaria solani*

**FIGURE 6:**
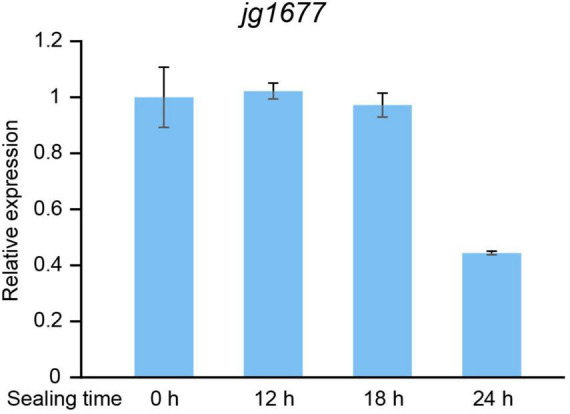
Expression levels of jg1677 of samples collected in plastic bags for different times at room temperature. qPCR detection of jg1677 expression for different sealing times. Data are shown as the expression of jg1677 compared to control. Error bars represent SD.

From the field to the laboratory, the collected leaves are usually kept in plastic bags for about 12 h at room temperature. To determine if the period of detachment of samples from potato plants interferes with the expression of jg1677, we mimicked the sampling process and tested the expression levels of jg1677. We started by infecting potatoes with *A. solani* and collecting the infested leaves after 24 h. After that, the isolated leaves were sealed in plastic bags and stored at room temperature, collected samples after 12, 18, and 24 h, respectively, and then used for assessing the expression levels of jg1677 by qPCR assay. There was less than a 2.5-fold difference in jg1677 expression levels between samples stored for 12, 18, and 24 h compared to control samples, according to the results. The expression of sealing 12 h is 102.2% of sealing 0 h, sealing 18 h is 97.17% of sealing 0 h, and sealing 24 h is 44.37% of sealing 0 h (Figure 6). This result indicated that it is viable to use the jg1677 transcript for pathogen detection in detached potato leaves. Transcripts of jg1677 were stably expressed in infested isolated leaves and were suitable for field collection and detection.

## Discussion

In this study, we developed an RNA-based method for sensitive and specific detection of *A. solani*. Previously, detection of *A. solani* was usually performed by conventional PCR amplification, morphological identification of the strain, and LAMP assay, which were developed based on genomic DNA (Rodrigues, 2009; Bock et al., 2010; Khan et al., 2018). But the sensitivities of these methods cannot meet the need during the latent period of EB (Leiminger, 2009; Latorse et al., 2010; Khan et al., 2018). Because of a lack of proper transcript information that is stably expressed at a high level under a wide range of conditions, it is difficult to ensure the specificity and sensitivity of RNA-based assays (Di Gaspero et al., 2022; Valmonte-Cortes et al., 2022). So, RNA-based methods have not been adopted in past studies. The breakthrough in this study was the identification of a gene, jg1677, that is specific and stably expressed in *A. solani*. By using 22 potato pathogens including *A. solani*, we verified that jg1677 is specific for *A. solani* (Figure 2) and can be used to detect EB. This method can detect as few as one spore infesting potato leaves. When compared to DNA detection, RNA detection proved to be more sensitive and efficient (Figure 3A). The application of this technology can greatly aid in pathogen detection and control of disease epidemics, as well as contribute to food production and food safety.

Methods used for EB detection were generally morphological or DNA-based tests (Rodrigues, 2009; Bock et al., 2010; Khan et al., 2018). Morphological testing is costly and laborious and is not the first choice for pathogen detection (Rodrigues, 2009). In response to this problem, subsequent researchers have developed DNA-based assays that greatly improve the efficiency while also ensuring the accuracy of pathogen detection (Tymon et al., 2016). The most sensitive DNA-based method is qPCR, which is up to thousands of times more sensitive than conventional assays (Tymon et al., 2016; Khan et al., 2018). In this study, we developed a qPCR assay based on RNA transcripts. We extracted RNA from the sample and reverse transcribed it into cDNA, which was diluted 20-fold before being tested using qPCR. Similarly, we extracted DNA from the sample and subjected it to qPCR after diluting the same multipliers. It was found that the jg1677 transcripts were up to 1,295-fold more abundant than that of jg1677 DNA (Figure 3A). This result confirmed that the RNA-based method is far more sensitive than the DNA-based method.

Stable gene expression is a prerequisite for the accuracy of the assay (Vandesompele et al., 2002), and changes in the external environment usually alter gene expression (Bennett et al., 2008), so experiments were conducted to investigate the effect of external temperature changes on jg1677 gene expression. The expression of jg1677 increased with increasing temperature, peaking at 25°C, and then began to decrease as the temperature increased further (Figure 4). Despite the fact that temperature changes affected jg1677 expression, we were still able to clearly detect the presence of *A. solani* in the experiment, indicating that changes in external temperature had no effect on the accuracy of our assay. Potatoes are grown in a large area with varying geographic and climatic conditions in the world, with temperatures ranging from 15 to 33°C (Ingram and McCloud, 1984). Although the ideal temperature for *A. solani* growth is around 28–30°C, spores of *A. solani* could be transmitted latently at any time. jg1677 could be highly transcribed in a temperature range of 15–35°C, which includes the growth temperature of potatoes and *A. solani*, and is suitable for general pathogen detection. This result eliminates the possibility of inaccurate results due to temperature changes during the testing process, as well as demonstrates the stability of our testing method.

It is difficult to avoid transporting samples from the field to the laboratory in plastic bags during the EB detection process. It should be determined whether this process has an effect on the stability of jg1677. Therefore, we compared the expression levels of jg1677 with the samples stored in plastic bags for different times, and the results showed that there was less than a 2.5-fold difference in jg1677 expression levels between the samples stored for 12, 18, and 24 h compared with control samples (Figure 6). In other words, transporting samples in plastic bags from the field to the laboratory after sampling has no effect on the accuracy of the assay results within 24 h, which is sufficient time for sampling from field to lab. This means that our testing methods do not need to be rushed after sampling and can be easily preserved.

*Alternaria solani* spores are spread primarily by air to healthy potato leaves (Dhaval et al., 2021; Padol Hrishikesh et al., 2021), and their rapid spread causes serious damage (Suganthi et al., 2020). Therefore, early detection of the pathogen upon the presence of EB in the field is critical for food production, the earlier an infestation is detected, the easier it is to control disease spread (Suganthi et al., 2020). RNA-based qPCR is more effective than morphological examination and DNA-based detection methods for detecting pathogen presence during the latent period of EB (Figures 3A, 5). This strategy enables sensitive, rapid, and specific detection of EB in the early stages of infestation in agricultural production activities, facilitating the prevention and timely control of this disease’s epidemic.

## Data availability statement

The original contributions presented in this study are included in the article/Supplementary material, further inquiries can be directed to the corresponding authors.

## Author contributions

LZ, JZ, and ZN conceived and designed the experiments and analyzed the data. ZN, PY, JW, YP, MT, DZ, and ZY performed the experiments. LZ and JZ wrote the manuscript. All authors reviewed the manuscript.

## Footnotes

1
http://www.ncbi.nlm.nih.gov/
