# Supplementary material for: Detection of Alternaria solani with high accuracy and sensitivity during the latent period of potato early blight
Source: Front Microbiol. 2022 Sep 23;13:1016996. doi: 10.3389/fmicb.2022.1016996 (PMC9537451; doi:10.3389/fmicb.2022.1016996)
Supplement: Supplementary file 1 [file Data_Sheet_1.docx]

**Supplementary Figure S1**


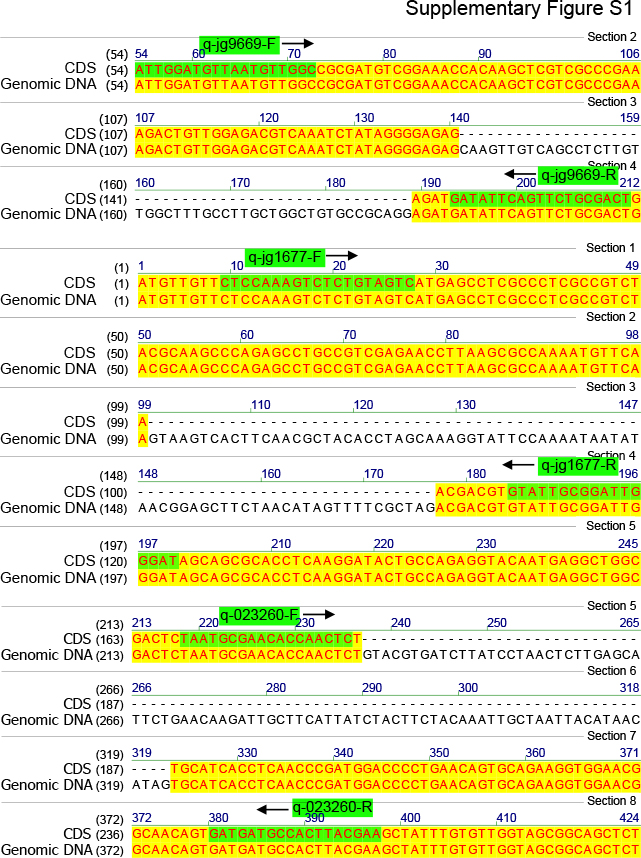


**Supplementary Figure S1**

Primers for jg1677, jg9669, and gene 023260 were designed based on genomic DNA and cDNA of *A. solani*. All primers can be matched exactly to genomic DNA and cDNA.

**Supplementary Table S1 Top ten gene sequences in transcript abundances**

| Gene | Sequence |
| --- | --- |
| jg9756 | ATGTCTGCTCCCAACGCCAACACTCCCAACGAGGGCATCGTCGGCCAGGCCGTCAACTCTGTCAAGAACGCCGCCAACTACGTCTCCGAGACCGTCCAGGGCTCCACCGCCGAGGCCAAGAAGGAGGCCAACAAGGAGCAGGCCAAGGGCAACGTCCCCGGCCAGGACTCCATCACCGACCGTGCCTCCGGTGCCCTCAACGCTGCTGGCGAGAAGCTCAACCAGGAGAAGCACGATGGCTCCGCCGAGGCCAACAAGCGCTCCATCTAA |
| jg9669 | ATGAGATTCTCTACGATCTTTGCCGCCGCTGGCCTTGTTATTGCAGCTACTGCATTGGATGTTAATGTTGGCCGCGATGTCGGAAACCACAAGCTCGTCGCCCGAAAGACTGTTGGAGACGTCAAATCTATAGGGGAGAGAGATGATATTCAGTTCTGCGACTGTTGCGCGGCGGGTGGATCTTGCTACAATCATTGCTGA |
| gene015970 | ATGAGCGAAATTCCCAAGGAGCAATGGGCGCAGGTCATCGAGAAGACCGGTGGACCCGTCGATTACAAAAAGATTCCCGTCCAGATGCCCGGTCCGGATGAGGTCCTCGTCAACATCAAGTACAGTGGTGTGTGCCACACAGATCTCCACGCTCTGAACGGCGACTGGCCATTGTCTACCAAACTGCCTCTTGTCGGCGGTCATGAAGGTGCCGGTGTTGTAGTCGCACGAGGCGAACTTGTCACAGATGTGGAGATTGGCGACTATGCTGGTGTCAAATGGCTAAACGGATCCTGCATGGCCTGTGATTACTGTCAGCAAGCTGACGAACCTCTTTGCCCAAAGCCTCTTCTCTCTGGATACACTGTCGACGGCACATTCCAACAATACTGCATCGCAAAAGCTGCCCACATCGCACGCATTCCCAAGGAGTGTGATCTTGCCGCCATTGCTCCGGTTCTGTGTGCCGGTATCACAGTCTACAAGGGCCTGAAAGAGTCGGGCGTCAAGCCCGGCCAATTCGCTGCCATTGTCGGTGCTGGTGGTGGTCTTGGTTCGCTTGCCTGCCAGTACGCAAAGGCAATGGGTGTACGAACAATCGCCATCGACACAGGTGACGAGAAGAGGAAGATGTGCACTGAAGACCTTGGTGCCGAGGCCTTTATTGATTTCTCCACGAGCAAGAACCTCGTCGCCGACGTACAGAAGGCAACGCCCGATGGCCTTGGTCCCCACGTTGTCATCCTTGTCGCCGTCAACGAGAAGCCCTTCCAGCAAGCTGCCGAGTACGTCCGCCCACGCGGTACGGTCATCTGCATTGGTCTTCCCGCTGGCGCCTATCTGAGGGCACCTGTTTTCGAGACGGTCCTCAGGATGATTAGGATCCAAGGTTCGTATGTCGGTAACCGCAAGGACAGCTCAGAGGCTATTGAGTTCTTCCGCCGTGGCTTGATCAAGGCCCCCTACAAGGTCGTTGGTATGTCTGAGCTCCAGATGGTCTATGACAAGATGAACAAGGGTGCCATTGTTGGGCGTTATGTCCTTGATACGTCGAAGTAG |
| gene087690 | ATGACATCTGTAAAGCTCACAACCCCCCAGACGGGCGAGTACGAGCAGCCCACCGGACTCTTCATCAATAATGAGTTCGTAAAGGCCGTTGACGGCAAGACCTTTGACGTCATCAACCCAAGTACCGAGGAGGTCATCTGCAGTGTTCAGGAGGCCACCGAGAAGGATGTCGACATTGCTGTCGCCGCCGCCCGCAAAGCCTTCAACGGCCCATGGCGGAAGGAGACACCCGAGAACAGAGGAAGGCTGCTCACTAAGCTCGCCGACCTTTTCGAGAAGAATGCCGACCTTATTGCCGCTGTCGAGGCTCTCGACAACGGTAAGGCCTTCAGCATGGCCAAGAACGTCGACGTACCCTCAGCCGCTGGTTGCCTGAGGTACTACGGAGGTTGGGCCGACAAGATTGAGGGCAAGGTCGTCGACACATCACCTGACACCTTCAACTATATCCGCAAGGAGCCTGTTGGTGTTTGCGGTCAAATCATTCCATGGAACTTCCCCATTCTCATGTGGGCATGGAAGATTGGCCCTGCCATCGCCACTGGTAACACTGTTGTCCTGAAGACTGCTGAGCAGACACCCCTCTCCGCATACATTGCCTGCACACTTATCAAAGAGGCCGGTTTCCCACCAGGTGTTATCAACGTCATCACTGGTTTCGGAAAGATTGCCGGTGCTGCCATGTCCGCTCACATGGACATTGACAAGATTGCCTTTACTGGTTCAACCGTTGTCGGTCGTCAGATCATGAAGTCTGCCGCTGGCTCCAACCTGAAGAAGGTCACTCTTGAGCTTGGAGGCAAGAGTCCCAACATTGTCTTTGCCGACGCAGACATCGACGAGGCTATCCACTGGGTCAACTTTGGTATTTACTTCAACCACGGCCAGGCTTGCTGTGCCGGTTCGCGTATCTACGTTCAAGAGGAGATCTACGACAAGTTCATCCAGCGCTTCAAGGAGCGGGCTGCTCAGAACGCTGTCGGTGACCCATTCGCCGCGGATACCTTCCAGGGTCCTCAGGTCTCGCAGCTCCAGTTTGACCGTATCATGGGCTATATCGAGGAGGGTAAGAAGTCTGGCGCGACCATCGAGACTGGTGGCAAGCGCAAGGGCGACAAGGGCTACTTCATCGAGCCCACAATCTTCTCCAACGTAACCGAGGACATGAAGATCCAGCAAGAAGAGATCTTCGGCCCCGTGTGCACAATCTCAAAGTTCAAGACAAAGGCCGATGTCATCAAGCTCGGCAACAACAGCACATACGGTCTTGCTGCCGCCGTCCACACATCCAACCTGACCACTGCCATTGAGGTTGCCAACGCGCTCCGTGCGGGAACCGTCTGGGTCAACTCCTACAACACGCTTCACTGGAGTTTGCCCTTTGGGGGGTATAAGGAGTCGGGGCTAGGTCGTGAGTTGGGTGAATCGGCGTTGGAGAACTACATCCAGACCAAGACGGTATCCATTCGTCTTGGCGATGTTCTGTTCGGTTAG |
| jg1193 | ATGGTTGCCATCGCTCGCTCCTTCGGCGCCGCCCGCGTGGCCGCCCGCGGCTTCTCCAACGCTGCCCGCCAGCCCCAGCAGAGCACCCTCGTCGCTGCCCGCAGTGCCTTCCGCAACAACGCCGCCCGCAACGTCATCCAGAAGCGTGGCATCGTCGCCGAGTCCACCGCTGCCGCCATGGTCGCTGCCGCCAAGATCCAGGGTGCCGGTCTCGCTACCATCGGTCTTGCCGGTGCCGGTGTTGGTATCGGAACGGTTTTCGGCGGTCTCATCCAGGGTGTTGCCCGCAACCCCTCCCTCAGGGGTCAGCTCTTCCAGTACGCCGTTCTCGGTTTCGCCTTCGCTGAGGCCACTGGTCTTTTCGCGCTCATGATGTCCTTCTTGCTCCTCTACGTCGCATAG |
| gene032980 | ATGACGATTCCAGATGAGGTCGACATCATTGTGTGCGGAGGTGGTTCGTGTGGTTGTGTCGTGGCTGGACGTCTCGCCAATCTCGACCACAATCTCCAGGTCCTCTTGATTGAGGCTGGAGAGAACAACCTCAACAACCCATGGGTTTTCCGTCCGGGTATCTACCCGAGGAATATGAAGTTGGACAGCAAGACTGCCACCTTCTACTACTCTCGTCCCTCGGAGTGGCTCGATGGCCGCCGAGCTGTTGTGCCTGTGGCACACATCCTCGGAGGTGGATCTTCCATCAACTTCATGATGTACACCCGTGCCTCGGCTTCCGACTACGACGACTTCCAAGCCAAGGGATGGACCACCAAGGAGCTCATCCCTTTGATGAAGAAGCATGAGACCTACCAGCGCTCCTGCAATAACCGTGAAATTCACGGATTTGAGGGACCCATCAAGGTTTCTTTCGGCAACTACACCTACCCGATCAAGGAGGACTTCCTGCGTGCCACTGAAACTCAGGGCATTCCGACCACCGACGACCTCCAGGACTTGACCACTGGACACGGTGCTGAGCACTGGCTCAAGTGGATCAACCGCGACACTGGACGTCGATCTGACTCCGCTCACGGTTACATTCACAGCACTCGCGCCGTTCACCAGAACCTGCACCTCCTCACTAGCAACAAAATCGACAAGGTCATTCTCGAGGGAGACCGCGCTGTTGGCGTCAAGGTTGTCCCAACTAAGCCTCTCCATGCCGAGCAGCAACGGTCCCGGATCATCAGGGCCCGCAAGCAGATCATTGTTTCTGGTGGTACTCTCAGCTCTCCTCTCATCCTTCAGCGCTCTGGTATCGGTGACCCAGAGAAGCTCCGCAAGGCCGGCGTCAAGCCTCTTGTCGACCTTCCTGGTGTTGGTCTCAACTTCCAGGACCACTACTTGACGTTTGCCACCTACCGCGCTAAGCCTCACGTTGAGTCGTTTGATGACTTCGTTCGTGGTGACCCCAAGGTCCAGGAGCAGGTCTTCAATCAATGGAACATCAACGGCACTGGTCCACTCGCTACCAATGGTATCGAGGCTGGTGTTAAGATCCGACCCACCGAGGAAGAACTGAACATGATGGACAGCTGGCCATGCAAGGAGTTCCGCTCCGGCTGGGATTCTTACTTCAAGAACAAGCCCGACAAGCCTGTTATGCATTATTCGGTCATTGCTGGTTGGTATGGTGACCACATGGTTATGCCACCAGGCAAGTTCTTCACCATGTTCCACTTCCTCGAGTACCCGTTCTCGCGTGGCTCTACGCACATTGTCTCTCCCAACCCATACGAGGCACCCGACTTTGACGCTGGTTTCATGAACGACAAGCGCGACATGGCTCCTATGGTCTGGGCTTACATCAAGTCTCGTGAGACTGCCCGCCGCATGGACGCTTACGCCGGCGAGGTGCAGGCAATGCATCCCTACTACGCCTTTGACAGTCCGGCCCGTGCCAATGACCTCGACCTTCTTACCACCAACCAGTACGCTCTTCCTGGTAACCTCTCGGCTGGTATTCAGCACGGATCTTGGACGTCTCCAGTCAAGAAGGGCCGCGCTCCCGAGCCCAACTTCTTGAACTCGAACTGCCAGGAGATTCATGAGGACCTCCAATACTCCAACGAGGATATCAAGCACATTGAGGACTGGGTCAAGCGTCACGTTGAAACCACATGGCACTCTCTCGGTACCTGTTCCATGGCCCCCAGGAACGGCAACTCCATCGTCAAGCACGGTGTCCTTGACGAGCGCCTCAACGTCCACGGTGTCAAGGGTCTCAAGGTTGCCGATCTGTCCATCTGCCCCGACAACGTCGGTTGCAACACATACTCGACTGCGCTCCTCATTGGAGAGAAGTGTGCAATGCTTACGGCTGAGGACTTGGGCTACAGCGGCAGCGCTTTGGACATGAAGGTCCCCAACTACCACGCTCCACGGGAGATTGCTGGTTTGTCGCGCTTGTAA |
| jg9792 | ATGCAGATCTTCGTCAAGACCCTCACGGGCAAGACCATCACTCTCGAGGTCGAGAGCTCCGATACCATCGACAATGTCAAGTCCAAGATCCAGGACAAGGAGGGCATTCCCCCGGACCAGCAGCGTCTGATCTTCGCCGGAAAGCAGCTCGAGGATGGCCGCACCCTTTCGGACTACAACATCCAGAAGGAGTCGACCCTCCACTTGGTCCTCCGCCTTCGTGGTGGTATGGCCAAGAAGCGCAAGAAGAAGGTCTACACCACCCCCAAGAAGATCAAGCACAAGCGCAAGAAGACCAAGCTAGCTGTCCTCAAGTACTACAAGGTCGACGGTGACGGCAAGATCGAGCGTCTTCGCCGCGAGTGCCCCCAGCCCGAGTGCGGTGCTGGTGTCTTCATGGCCGCCATGCACAACCGCCAGTACTGCGGAAAGTGCCACTTGACCTACGTCTTCGACGAGGCCAAATAA |
| gene032230 | ATGGCCCGCACTAAGCAGACCGCCCGCAAGTCCACTGGTGGCAAGGCTCCCCGCAAGCAGCTCGCATCCAAGGCAGCTCGCAAGTCCGCACCGTCAACCGGTGGTGTCAAGAAGCCTCACCGCTACAAGCCCGGAACCGTCGCTCTCCGTGAGATCCGTCGCTACCAGAAGTCGACTGAGCTCCTCATCCGCAAGCTGCCCTTCCAGCGTCTTGTCCGTGAGATTGCCCAGGACTTCAAGTCGGATCTCCGCTTCCAGTCTTCTGCCATCGGCGCTCTCCAGGAGTCCGTCGAGGCCTACCTCGTCTCCCTCTTCGAGGACACCAACCTCTGCGCCATCCACGCCAAGCGTGTCACCATCCAGAGCAAGGACATCCAGCTCGCCCGCCGCCTCCGCGGTGAGCGTGGTTAA |
| jg1677 | ATGTTGTTCTCCAAAGTCTCTGTAGTCATGAGCCTCGCCCTCGCCGTCTACGCAAGCCCAGAGCCTGCCGTCGAGAACCTTAAGCGCCAAAATGTTCAAACGACGTGTATTGCGGATTGGGATAGCAGCGCACCTCAAGGATACTGCCAGAGGTACAATGAGGCTGGCCAGGCGCTAGGTGGCCAGGCGTGCCGCAGGGCTTCTCCTTGCAACACTCAGGGTAATGGGTGCATCCTGAACATTGAAAATTATGAGGGCGGTTGGTATGCCAACTGCAGCTAA |
| gene023260 | ATGAAGATCAACATCCTTCTTGCAAGCTTTGCGGCCTGTGCGCCACTTGTCGCAGGCCAGTTCTCTTGCCAATGCCAGGCTATTCCTAGGCCTGGTACAAATGGACGGCCTGCTTTCTATGCTGGTGTCGGCTGTACCGAAGTTCGTGGAGTGCTCTGTTTTGACTCTAATGCGAACACCAACTCTTGCATCACCTCAACCCGATGGACCCCTGAACAGTGCAGAAGGTGGAACGGCAACAGTGATGATGCCACTTACGAAGCTATTTGTGTTGGTAGCGGCAGCTCTTGCTCCTCTTAA |
